# Newborn Screening Program for Spinal Muscular Atrophy in the Campania Region (Italy): Current Limitations and Potential Perspectives

**DOI:** 10.3390/ijns11030064

**Published:** 2025-08-17

**Authors:** Adelaide Ambrosio, Tiziana Fioretti, Barbara D’Andrea, Lucia Pezone, Ilaria Bitetti, Carmela Di Domenico, Sabrina Vallone, Valeria Maiolo, Angela Cioce, Mariano Giustino, Antonio Varone, Gabriella Esposito

**Affiliations:** 1 CEINGE Advanced Biotechnologies Franco Salvatore, s.c.a r.l., 80145 Naples, Italy; ambrosioa@ceinge.unina.it (A.A.); fioretti@ceinge.unina.it (T.F.); dandrea@ceinge.unina.it (B.D.); pezone@ceinge.unina.it (L.P.); didomenico@ceinge.unina.it (C.D.D.); sabrina.vallone99@gmail.com (S.V.); giustino@ceinge.unina.it (M.G.); 2 Neurology Unit, Santobono-Pausilipon Children Hospital, 80122 Naples, Italy; i.bitetti@santobonopausilipon.it (I.B.); a.varone@santobonopausilipon.it (A.V.); 3 Integrated Care Department of Laboratory and Transfusion Medicine, University Hospital Federico II, 80131 Naples, Italy; a.cioce06@gmail.com; 4 Department of Molecular Medicine and Medical Biotechnologies, School of Medicine, University of Naples Federico II, 80131 Naples, Italy; valeriamaiolo1999@gmail.com

**Keywords:** spinal muscular atrophy, newborn screening, carrier screening, targeted therapy

## Abstract

Three targeted therapies are currently available for spinal muscular atrophy (SMA), which have dramatically changed the natural history of this severe and potentially fatal disease. More than 95% of SMA cases have a homozygous deletion of exon 7 of the *SMN1* gene. Disease expression mainly depends on the copy number of *SMN2*, a hypomorphic copy of *SMN1*. Many countries in the world have implemented newborn screening (NBS) programs for early identification and treatment of children with SMA. We herein present the first two-year results of the SMA NBS program in Campania, a region with one of the highest birth rates in Italy. Genomic DNA was extracted from dried blood spots (DBS) and peripheral blood. For DBS, the *SMN1* gene copy number was evaluated by quantitative polymerase chain reaction (qPCR) targeting *SMN1* exon 7 and a reference gene (*RPP30*). In positive newborns and their parents, *SMN1*/*SMN2* copies were evaluated by multiplex ligation probe amplification (MLPA). We analyzed 77,945 newborns and identified 11 positive children. Six patients had 2 copies of *SMN2*, but only one showed severe SMA-related signs at birth. Eligible newborns were treated with gene therapy within 20 days of birth. Notably, qPCR failed to amplify the reference *RPP30* gene in 10/77,945 DBS. Despite this limitation, we observed that about 1/40 DBS had ΔCt values consistent with the presence of one *SMN1* copy. The semi-automated procedure used for SMA NBS showed excellent performance in detecting the presence of homozygous deletion of *SMN1* exon 7, with the exception of a few cases with the absence of amplification of the reference gene. By solving this limitation, the screening procedure has the potential to detect heterozygous carriers of the *SMN1* deletion and, consequently, identify families at procreative risk of SMA.

## 1. Introduction

Spinal muscular atrophy (SMA) is a neuromuscular disease characterized by progressive muscle weakness and atrophy due to degeneration and loss of motor neurons in the anterior horn of the spinal cord [1]. The incidence of SMA is approximately 1 in 3900–16,000 live births in Europe [2,3], and the carrier rate is about 1/40–50 in most populations. If untreated, SMA is the leading genetic cause of infant death worldwide [1,4].

Four major SMA subtypes, ranging from type 0 (prenatal onset) to type 4 (adulthood onset), have been recognized, based on disease severity and age of onset [5]. More than 60% of SMA patients developed SMA type 1 [4]. In the natural history of the disease, children with SMA type 1 are unable to sit, maintain head control, or eat independently; they require early mechanical ventilation, with mortality usually caused by respiratory failure [1,5].

SMA is primarily associated with pathogenic variants in the *Survival Motor Neuron 1* (*SMN1*; OMIM *600354), located on chromosome 5q13. This gene has a paralog, *SMN2* (OMIM *601627), and is closely related to the *NLR family apoptosis inhibitory protein/BIRC1* (*NAIP*) gene (OMIM *600355), both also located on chromosome 5q13. The *NAIP* gene lies about 15 kb downstream of *SMN1*, while *SMN2* is about 875 kb upstream of *SMN1* and shares > 99.9% sequence identity with it. Both *SMN1* and *SMN2* encode the molecular chaperone SMN, which is crucial for the assembly of RNA and proteins in ribonucleoprotein complexes [6].

A key difference between *SMN1* and *SMN2* lies at nucleotide position c.840 in exon 7. In *SMN1*, this position is occupied by a cytosine (C), while in *SMN2*, it is a thymine (T). This difference disrupts an exonic splicing enhancer in *SMN2*, leading to the skipping of exon 7 in about 90% of transcripts that generate an unfunctional gene product [7,8].

Approximately 95% of SMA cases are caused by the biallelic absence of the *SMN1* c.840C nucleotide, either due to deletions encompassing at least exon 7 of *SMN1*, or through gene conversion events that convert *SMN1* into *SMN2*. The remaining ~5% of patients are compound heterozygotes, in whom one *SMN1* allele is absent-either deleted or converted–while the other carries a different pathogenic variant [9]. In contrast, the biallelic loss of *SMN2* exon 7 or *NAIP* exon 5 is a relatively common polymorphism in healthy subjects [8].

Alleles with multiple copies of *SMN1*, *SMN2*, and *NAIP* are also found in the general population [7]. The presence of two or three copies of *SMN1* on the same allele is frequent and can mask about 3% of the heterozygous carriers of *SMN1* deletion/conversion, leading to the silent SMA carrier status [10]. In SMA patients, the number of *SMN2* copies affects the disease’s onset and severity [7]. Patients with ≤2 *SMN2* copies may experience an infantile-onset form; three *SMN2* copies are often associated with intermediate severity phenotypes; and ≥4 copies generally indicate a milder, later onset, disease. Biallelic loss of *NAIP* is a negative disease-severity predictor, especially in patients with ≤3 *SMN2* copies [11].

In SMA patients, the *SMN2* polymorphism c.859G>C is a positive disease-modifier. The *SMN1* c.840C>T pathogenic conversion, in homozygous individuals and compound heterozygotes with a deleted *SMN1* allele, can also be considered predictive of a less severe phenotype even in patients with ≤2 *SMN2* copies [12].

Recent advances in SMA therapeutics have produced and approved 3 specific drugs. Nusinersen (Spinraza^TM^, Biogen/Ionis Pharmaceuticals, Cambridge, MA, USA) and risdiplam (Evrysdi, Genentech/Roche, South San Francisco, CA, USA), an antisense oligonucleotide and a small molecule, respectively, have been successfully used to modulate inclusion of exon 7 in *SMN2* transcripts. Onasemnogene-abeparvovec-xioi (Zolgensma^TM^, Novartis Gene Therapies, Bannockburn, IL, USA) is a gene-therapy drug that consists of self-complementary adeno-associated virus serotype 9 capsids containing the *SMN1* transgene [5,13]. Their use is changing the natural course of the disease and is generating new SMA phenotypes that require new classifications [14,15].

Since available treatments are especially effective when they are given before SMA symptoms appear, a newborn screening (NBS) program for early, often presymptomatic, diagnosis of SMA has been promoted worldwide to start the most appropriate therapy as early as possible [16,17].

Currently, the Italian health regulatory agency allows treatment with gene therapy for children with SMA up to 13.5 kg and presymptomatic babies with ≤3 *SMN2* copies, whereas nusinersen and risdiplam can be administered at all ages to presymptomatic SMA patients with one to four *SMN2* copies. Similarly, for symptomatic newborns, the treatment decision depends on the severity of the condition and the *SMN2* copy number [15]. As a result of this change in treatment policy, many more Italian regions have implemented NBS programs for early identification of SMA children [18,19].

Despite the birth rate in Italy having dropped significantly in the last 10 years, in 2022, compared to 2021, births have slightly increased in the Campania region (about 45,000 newborns/year), which started an NBS program for SMA in April 2023. In the same diagnostic center, dried blood spots (DBS) collected for the mandatory screening of inborn errors of metabolism (IEM) were also analyzed to detect, by real-time polymerase chain reaction (PCR), the absence of *SMN1* exon 7, which represents a highly specific molecular marker of SMA.

We herein report results of the first two years of NBS activity for SMA in the Campania region, which represents a clear example of healthcare collaboration aimed at the diagnosis, treatment, follow-up, and prevention for patients affected by this severe, but currently treatable, inherited disease and their families.

## 2. Materials and Methods

Since 3 April 2023, infants born in any of the 54 birth centers in Campania have been screened for SMA as a part of a dedicated NBS pilot project, which was approved by the Ethics Committee of the Santobono Children’s Hospital of Naples. The test was voluntary and required the signing of an informed consent.

At the birth centers, after informed consent was signed, neonatal blood spots for SMA screening were collected on a filter paper card (EBF 903 Multiple-Part Neonatal Card, Expertmed srl, Verona, Italy) simultaneously with those for the mandatory IEM, at 48 or 72 h of the newborn’s life and then sent to the Regional Reference Center for NBS (CEINGE–Biotecnologie Avanzate Franco Salvatore s.c.a r.l., Naples, Italy) [20]. The staff of the birth centers recorded personal information and contact details of newborns and mothers on the filter paper card and the so-called “Campania Neonatal Screening (SNC)” platform (Kelyon s.r.l., Naples, Italy), a digital platform that enables multi-center management of information flows relative to the NBS programs active in Campania. On the platform were also recorded newborn and maternal clinical information, such as ongoing drug therapies/treatments, diseases, transfusions of blood components, as well as any sample non-conformities. DBS arrived at the reference center at 4–6 days of the newborn’s life; the SMA laboratory received and analyzed only DBS of newborns for whom consent was collected. SMA screening testing was performed within 1–6 days after, and results were immediately shared with the birth centers and the reference clinical center for the therapy through the SNC platform. After testing, DBS are stored at room temperature for 1 year and then destroyed, as indicated in the informed consent. A complete integrated workflow, previously validated by other Italian screening centers [18,19], was used to extract DNA from DBS (Eonis DNA Extraction kit, Revvity, Waltham, MA, USA), and to perform a real-time PCR-based assay for the detection of the exon 7 *SMN1* gene (Eonis SMA kit, Revvity), which was carried out with a QuantStudio Dx Real-Time PCR instrument (Thermo Fisher, Waltham, MA, USA) [19]. The PCR assay targets the *SMN1*:c.840C nucleotide in exon 7, and *RPP30* as an internal amplification control using sequence-specific primers and Taqman probes. Analysis and interpretation of results were performed using Eonis analysis software (Revvity). A Ct threshold cutoff of 31.2 for *SMN1* was established to discriminate between presumptive positive and negative results. Positive results were confirmed on a second DBS of the same card. Statistical difference between true positives (carriers) and true negatives (normal) was performed by Student’s t-test with *p*-value < 0.05. To detect possible heterozygous SMA carriers in the population of newborns (unknown), we set a threshold of ΔCt > 0.05, a value above 2-fold the standard deviation plus the mean of the control carriers. Although our integrated platform allowed multiplexing of SMA and immunodeficiencies (SCID and XLA) in one assay [21], we extrapolated and analyzed only data relative to SMA.

Fresh blood samples of presumed positive infants and their parents were collected to perform the molecular test to confirm the screening results, and to determine the copy number of *SMN2* and *NAIP*. DNA was extracted, and a definitive molecular diagnosis was carried out by multiplex ligation probe amplification (MLPA; SALSA MLPA Probemix P021 SMA, MRC Holland). The presence of the *SMN2* positive disease-modifier c.859G>C was evaluated by allele-specific amplification. In brief, for each patient, 2 PCR reactions were set up, each containing the DNA sample, a reverse *SMN2* common primer (5′-TTCTTCCACACAACCAACCA-3′), and either the forward (c.859G: 5′-GGGTTTTAGACAAAATCAAAAACAAG-3′) or mutant (c.859C: 5′-GGGTTTTAGACAAAATCAAAAACAAC-3′) allele-specific primer, which differed only in their 3′ nucleotide. As an internal amplification control, exon 2 of the *HBB* gene was co-amplified.

The *SMN1* polymorphic variants c.*3+80T>G and *SMN1* c.*211_*212del, which are associated with *SMN1* gene duplication and “silent carrier” status, were detected using the AmplideX^®^ SMA Plus Kit* (Asuragen Inc., Austin, TX, USA).

Manual DNA extraction from DBS was performed using the Chemagic^TM^ DNA Cyto pure Kit (Revvity). Exon 1 of *RPP30* was amplified with the opportune primers (forward 5′-AACTGGAGGTAGAGACGGAC-3′; reverse 5′-CGAGACCCATCAGGAAA-3′) and sequenced.

## 3. Results

The study started on 3 April 2023. All 54 birth Centers of the Campania region adhered to the study. The compliance of families was 91% with respect to the overall number of DBS that were sent to the CEINGE Center for the mandatory IEM screening.

### 3.1. NBS Screening Results

From April 2023 to 31 March 2025, 77,945 children born in the Campania region underwent NBS for SMA. All samples were successfully analyzed, and no resampling or manual DNA extraction was required for *SMN1* analysis. Tests were repeated for less than 0.5% of children, mainly due to poor amplification of targets. No false positive results occurred. Since the SMA NBS is based on DNA analysis, transfusion of leukocytes, while uncommon, can provide a false negative result, which can, however, be verified by analyzing a different biological sample, such as saliva. In our cohort, no newborns underwent this or any other type of blood component transfusion.

**Figure 1:**
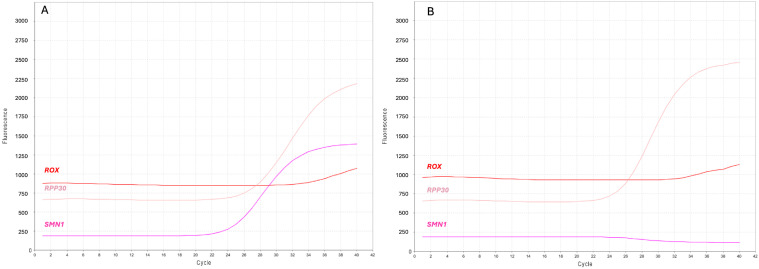
Screenshots of the real-time PCR curves indicating presumed positive SMA newborns. (**A**) Multicomponent plot from a negative DBS, and (**B**) a presumed positive newborn. *SMN1* (purple) and *RPP30* (pink) label the amplification curves of their respective targets; *ROX* (red) labels the constant fluorescent signal for sample normalization.

Overall, we identified 11 presumed positive samples, 9 females and 2 males, for which only amplification of the reference gene was obtained (Figure 1A).

The earliest diagnoses were made at 6 days of age (3 patients) and the latest at 10 days (1 patient), with a median age at diagnosis of 7 days. An additional affected male was diagnosed at 1 year of age, as the parents had not given consent for testing at birth.

**Figure 2:**
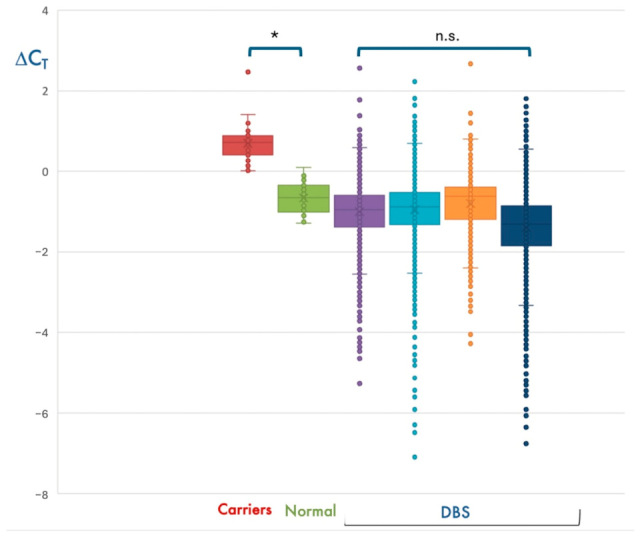
Detection of presumed SMA heterozygous carriers during NBS. Distribution of ΔCt values obtained for the ~30,000 newborns analyzed in the first 4 bimesters of the NBS program (DBS), in subjects with 2 *SMN1* copies (Normal), and in heterozygous deletion carriers (Carriers). As expected, most DBS have median ΔCt values compatible with 2 *SMN1* copies, which is the most represented genotype in the general population. * *p* < 0.05; n.s., not significant.

In most cases, *SMN1* amplification resulted in a threshold cycle (Ct) lower than that of *RPP30*, with a ΔCt = (Ct*SMN1*-Ct*RPP30*) < 0. However, about 1/35 DBS had a ΔCt > 0. Due to the genetic complexity of the 5q locus, we hypothesized that samples with ΔCt > 0 could be heterozygous carriers of the *SMN1* exon 7 deletion. Using the same automated procedure, we analyzed the DNA of 30 subjects who were previously diagnosed as SMA carriers and 30 controls with 2 *SMN1* copies. Interestingly, individuals with 2 *SMN1* copies had ΔCt < 0, whereas all heterozygous carriers had a ΔCt > 0, thereby supporting our hypothesis (Figure 2).

**Figure 3:**
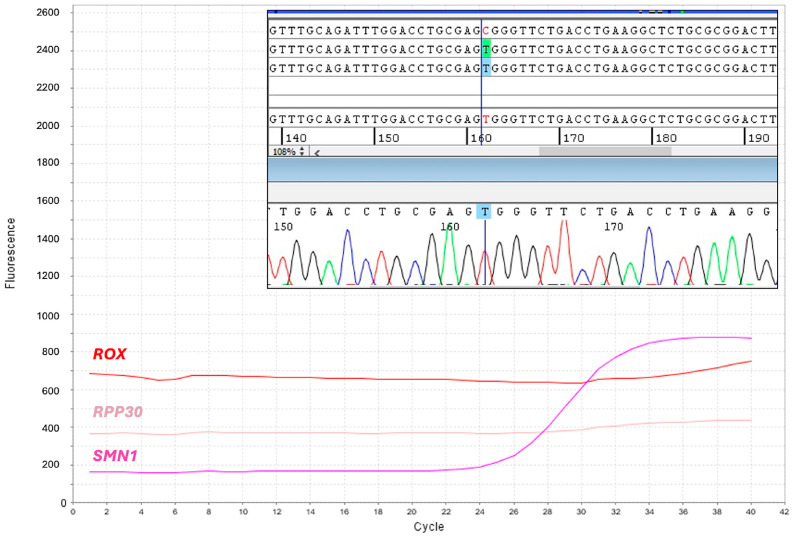
Identification of homozygotes for the *RPP30* c.32C>T polymorphism. Screenshot of the multicomponent real-time PCR plot shows absent amplification of the reference gene, *RPP30*. *SMN1* (purple) and *RPP30* (pink) label the amplification curves of their respective targets; *ROX* (red) labels the constant fluorescent signal for sample normalization. Panel inside the plot shows the electropherogram obtained from Sanger sequencing of the *RPP30* exon 1, which reveals homozygosis for the c.32C>T polymorphism.

For 10 DBS (1/7795), amplification of the *RPP30* reference gene failed. We found that loss of *RPP30* amplification depended on the c.32C > T polymorphism (dSNP rs41286916) that impaired probe hybridization, thereby causing a full allelic dropout in homozygotes, as demonstrated by amplification and sequencing of the *RPP30* exon 1 (Figure 3), which was performed following manual DNA extraction. Nevertheless, the *SMN1* amplification plot was present in these newborns, who were therefore considered presumptive negative for SMA and not further analyzed.

Heterozygotes for the *RPP30* exon 1 polymorphism escape detection; however, they should result in a higher Ct than that obtained for a homozygous wild type. No samples with amplification loss of both *SMN1* and *RPP30* were detected.

### 3.2. Molecular Confirmation of SMA and Evaluation of SMN2 Copy Number

Presumed positive SMA newborns identified through NBS were reported to the Neurology Unit of Santobono Pediatric Hospital, which informed the families about the disease and therapeutic options, referred infants to the first neurological assessment, and included them in a dedicated clinical care pathway [15]. Moreover, peripheral blood of the newborns and their parents was collected and sent to the CEINGE Center to perform the second-tier molecular test.

Genomic DNA extracted from blood leukocytes was analyzed by MLPA to determine the copy number of *SMN1*, *SMN2*, and *NAIP* in patients and their parents. Assessment of *SMN1*, *SMN2,* and *NAIP* copy number revealed 8 different genotypes in the 11 positive infants. The same test was also performed for an additional affected child, who had not adhered to the NBS, but had been admitted in December 2024 to the emergency room of Santobono Pediatric Hospital with symptoms suggestive of SMA; this child had a further different genotype in terms of *SMN1*, *SMN2,* and *NAIP* copy number (Table 1).

**Figure 4:**
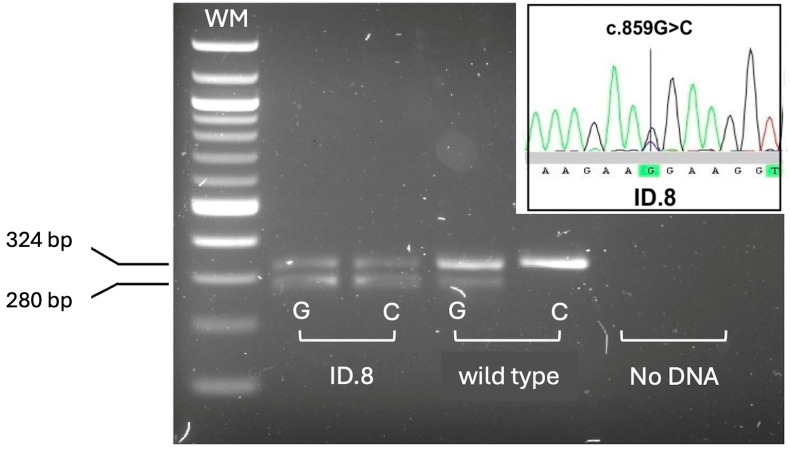
Rapid detection of the *SMN2* c.859G>C polymorphism by allele-specific amplification. Allele-specific amplification products detected by agarose gel electrophoresis. Only patient ID.8 showed the amplification product (280 bp) specific for the positive disease-modifier allele c.859C, at the heterozygous state. Wild type: homozygous control for the common c.859G allele. *HBB* exon 2 (324 bp) was co-amplified as an internal amplification control. WM: DNA molecular weight marker. The panel inside the gel shows the electropherogram obtained from Sanger sequencing of the *SMN2* exon 7, in ID.8 patient, confirming heterozygosis for the c.859G>C variant.

Ten positive newborns were homozygous for deletions removing the whole *SMN1* gene. Among these, 5 had two *SMN2* copies, a genotype relatable to severe SMA type 1; two children had three, and 3 had four *SMN2* copies. The remaining 2 positive newborns were compound heterozygous for *SMN1* deletions and the c.840C>T conversion, one with two *SMN2* copies, whereas the other had three *SMN2* copies. The presence of the conversion was confirmed by a further assay that allowed detection of *SMN1–SMN2* hybrid peaks, including those resulting from gene conversion events [22]. One male child with 4 *SMN2* copies carried the c.859G>C positive disease-modifier in *SMN2* (Figure 4).

Four infants with homozygous *SMN1* gene deletion and 2 *SMN2* copies also resulted homozygous for the deletion of the *NAIP* exon 5, a negative disease-modifier, two of which were symptomatic, but only one needed neonatal intensive care.

**Figure 5:**
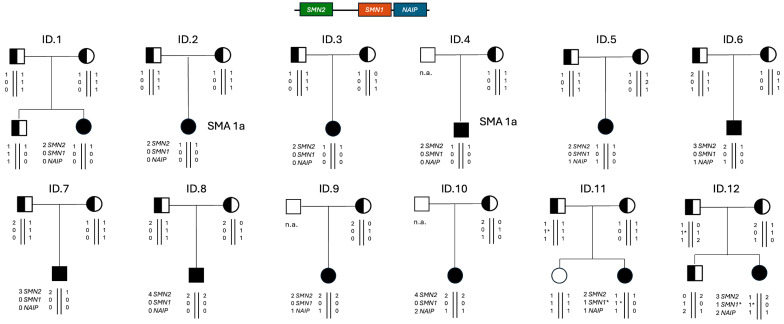
Pedigrees of the positive newborns’ families with the presumed genotype of each analyzed subject. SMA 1a indicates the prognosis in the absence of a molecular therapy, based on the *SMN2* copy number and presence of symptoms at birth. n.a., not analyzed. 1*, *SMN1* allele with the pathogenic c.840C>T conversion.

The *SMN1* genotype of positive SMA newborns was consistent with the genotype of parents that were indeed heterozygous carriers with one deleted/converted copy of *SMN1*, except for a mother with two *SMN1* copies. In this latter case, since grandparents or other relatives were not available for molecular testing, we were not able to discriminate whether this mother was a silent SMA carrier with a 2/0 *SMN1* genotype, or a de novo *SMN1* deletion occurred. Interestingly, this family was of African origin, a lineage in which the 2/0 genotype is quite frequent, and the mother was found to be heterozygous for the *SMN1* c.*3+80T>G and c.*211_*212del polymorphisms, which have been associated with the *SMN1* gene duplication, indicating a high risk of being a “silent” carrier of SMA [23]. In contrast, although the overall *SMN2* copy number was consistent between children and parents, in many patients, we were unable to precisely assess the phase of the *SMN2* copies and therefore determine the definitive genotype (Figure 5).

### 3.3. Management of Positive Newborns

The youngest positive newborns were referred to the Neurology Unit at the Santobono Children’s Hospital at 7 days (2 patients) and the oldest patients (2 infants) at 11 days, with a median age at the first neurologic visit of 9 days.

Eight of the 11 positive newborns did not present pathognomonic signs/symptoms of SMA at birth, 2 were paucisymptomatic with slightly hypoactive deep tendon reflexes and mild hypotonia, and 1 female patient presented severe hypotonia and required intensive care. During the first visit, blood samples were collected from the patients and parents to perform the confirmatory genetic test that also assessed the *SMN2* copy number [15].

Within 1–2 days from blood sampling, the results of the confirmatory genetic test were communicated to the parents in the context of a multidisciplinary genetic counseling session, to provide more detailed information on the expected prognosis, therapeutic options, and reproductive risk for the parents and their relatives.

A multidisciplinary team of specialists, neurologists, pediatricians, physiatrists, and other healthcare professionals counseled the parents and recommended the most appropriate treatment regimen. This was based on the SMA type/severity, for symptomatic newborns, on the *SMN2* copy number, for symptomatic and asymptomatic babies, and on the general health condition.

Nine patients had ≤3 *SMN2* copies and were eligible for gene replacement therapy, but, at the parents’ request, only 6 received the transgene. Five of these children, 3 asymptomatic, 1 paucisymptomatic and the only symptomatic patient, were treated within 22 days of life; the sixth patient, because of the detection of anti-AAV9 antibodies which made him ineligible for gene therapy, was initially treated with nusinersen at 11 days of life and, 2 months later, when the antibody titer became negative, the therapy was switched to onasemnogene-abeparvovec. The 5 patients that did not receive the onasemnogene-abeparvovec, 4 asymptomatic and 1 paucisymptomatic, were treated with risdiplam within 40 days of life. AAmong them were the 3 patients with 4 *SMN2* copies, and the patient with 3 *SMN2* copies and the *SMN1*:c.840C>T conversion.

In our settings, all the children identified by NBS were treated with one of the available therapies for SMA. The youngest infant was treated at 11 days, and the oldest patient at 40 days. Before treatment, a compound muscle action potential (CMAP) test of the ulnar nerve was recorded at baseline for all patients, who also underwent a rigorous clinical follow-up to detect possible signs of the disease. The follow-up procedure consisted of blood sampling and neurological examination that included standardized assessments of motor function and overall psychomotor development by the Children’s Hospital of Philadelphia Infant Test of Neuromuscular Disorder (CHOP-INTEND) scale and the Bayley III scale of infant and toddler development, Third Edition.

None of the treated babies, currently aged between 3 and 27 months, has had any therapy-related complications at the last follow-up. All the presymptomatic babies have had appropriate psychomotor development by acquiring the skills expected of healthy peers in an appropriate time frame, independently of the type of therapy administered; the two symptomatic newborns with 2 *SMN2* copies also responded well to the therapy, achieving much better psychomotor development than would be naturally expected for severe SMA forms. Notably, the first identified child, who was asymptomatic with 2 copies of *SMN2*, was treated with onasemnogene-abeparvovec and acquired walking before 12 months. In contrast, the child with 3 *SMN2* copies who was diagnosed at 12 months because he did not undergo NBS has had the worst outcome despite having started therapy immediately after diagnosis, but 6 months after the first signs of the disease appeared.

## 4. Discussion

In 2023, the number of live births in Campania was 42,925, about 3.5% less than in 2022, reflecting the overall negative trend in birth rates that has afflicted Italy from 2000 to today (−30%). This region represents over 11% of the national birth rate.

Based on the number of DBS that arrived at the CEINGE Center for the mandatory IEM screening, the compliance of families with SMA screening was 91% mainly due to the non-mandatory nature of the testing, as all the 54 regional birth centers adhered to the study. Screening results were shared with the birth centers through the SNC platform, which was implemented for the management of information flows obtained from the NBS programs that are active in this Italian region, namely IEM, hearing disorders, and SMA. In the current context of constant research and innovation, the SNC platform is one of the most advanced preventive medicine tools for newborns, and it is poised to become a model for all other regional administrations.

The main challenge in the screening organization was the coordination of the 54 regional birth and hospital centers, which, although they were already active in collecting and shipping DBS for mandatory screening, had to accept the burden of providing informed consent and collecting parents’ signatures. Obviously, if SMA NBS were to become mandatory, this phase could be less demanding. In any case, in view of a national law that will hopefully soon make SMA screening mandatory, all Italian regions must implement pilot programs or enter into agreements with regions that have implemented SMA NBS. No more SMA babies should be born in any Italian region without the opportunity to receive the early diagnosis and timely treatment they need to live a longer and hopefully healthy life.

Since the beginning of the pilot project to 31 March 2025, SMA NBS in Campania has covered 77,945 newborns. As with other Italian regions, the frequency of SMA in Campania is 1/6580–7085 [18]. Indeed, with the inclusion of a 1-year-old affected infant identified outside the NBS, there were 11 confirmed cases within the NBS cohort. Data analysis was based on the presence/absence of the amplification plot relative to the target *SMN1* gene, and the presence of the curve corresponding to the reference *RPP30* gene. No false positive results were obtained.

It is noted that homozygosity of the c.32C>T polymorphism in the *RPP30* exon 1 resulted in the lack of amplification of the reference gene, not of the *SMN1* target, in a significant proportion of samples (1/7795), which were considered atypical unaffected newborns. As we detected 10 of such atypical newborns, the minor allele frequency (MAF) of this polymorphic variant in our patient cohort should be about 0.022653. This evidence reveals a criticism of the screening methodology to be resolved promptly; indeed, the coexistence of homozygous *SMN1* defect and *RPP30* polymorphism would lead to total absence of amplification plot for both targets, making identification of a true positive SMA infant difficult. Currently, manual DNA extraction and amplification of the target and reference gene would be able to solve such a rare but still possible situation. This issue could be permanently circumvented by updating the amplification mix composition with a primer pair that does not anneal in a polymorphic region of the *RPP30* gene, a solution already proposed to the kit’s manufacturer. An alternative solution could be using other qPCR assays for the detection of the *SMN1* gene absence, which, however, would require at least the acquisition/adaptation of both the automated DNA extraction instrument and the data analysis software [24].

As the SMA real-time PCR assay simultaneously analyzes a target and a reference gene, a quantitative evaluation of data could be performed to detect subjects with one copy of *SMN1* by calculating the ΔCt. This calculation would have the potential to identify heterozygous carriers of *SMN1* deletion/conversion and provide parents and family members with the opportunity of carrier screening for future pregnancies. Based on the SMA frequency determined by our study, unaffected deletion carriers of SMA in our cohort should be about 1/42 individuals. Interestingly, most DBS resulted in a ΔCt = (Ct*SMN1*-Ct*RPP30*] < 0, whereas about 1/40 had a ΔCt > 0.1, similar to the results obtained for 30 known SMA carriers we analyzed with the same methodology. Unfortunately, we could not perform a confirmation test in any of the DBS with a ΔCt > 0.1, because we did not disclose the possibility of carrier identification to the parents in the informed consent. However, in the Campania region, based on the estimated SMA carrier risk assessed in this study, we expect that at least 25 couples/year are at risk of having affected offspring.

Moreover, since heterozygosity for the c.32C>T polymorphism increases the *RPP30*-related Ct, double heterozygotes with one *SMN1* copy and this polymorphism could escape detection, thereby affecting the overall carrier detection rate. Further studies need to validate our observation, which, however, can be considered to support the hypothesis that our NBS protocol is able to detect most SMA carriers with one *SMN1* copy, as already reported for other qPCR-based NBS for SMA [25,26]. Obviously, silent SMA carriers with a 2/0 *SMN1* genotype cannot be detected.

The second-tier test that was applied to the qPCR-positive newborns and their parents was MLPA. Although MLPA analysis could also be performed on DNA extracted from DBS, the quality of the result was not always reliable in determining the precise copy number of the *SMN2* gene. Therefore, to obtain a highly reliable result and to exclude possible errors during DBS sampling, we preferred to analyze a new blood sample from the newborn and their parents. MLPA methodology, which in our setting provide results in 1–2 days from the blood sampling, discriminates the deletion involving the entire *SMN1* gene (82% of our patients) from that limited to exon 7 and 8, or from the c.840C>T conversion (18%), which has a less severe impact on the residual gene expression and therefore is considered a positive disease modifier [27]. This latter information is relevant in the definition of the *SMN2* copy number, because when a confirmatory test targets only the c.840 nucleotide of both *SMN1* and *SMN2*, the copy of *SMN1* with the c.840C>T conversion is erroneously attributed to *SMN2*. This incorrect genotyping leads to overestimation of *SMN2* copies and impairs clinical classification and prognosis of such patients. MLPA also detects homozygous deletion of the *NAIP* gene, a negative prognostic marker of disease severity that we identified in 6 patients, 50% of whom (2 with 2 copies and 1 with 3 copies of *SMN2*) were symptomatic at birth. Lastly, this methodology also has the potential to detect copy number variants that affect the remaining exons 1–6 of the *SMN* genes [28].

## 5. Conclusions

It has been shown that SMA NBS coupled with early treatment is cost-effective compared with late treatment following clinical diagnosis and is dominant when societal perspective, caregiver burden, and treatment based on parental preference were considered [29]. Currently, the main goal of SMA NBS is to identify affected newborns to be treated promptly with the available therapies. All SMA children intercepted in the Campania region by NBS have received the appropriate treatment and are currently monitored in a close follow-up.

*SMN2* copy number is the main determinant of therapeutic decision, especially in the asymptomatic infants identified by NBS. Assessment of *SMN2* copy number requires quantitative methodologies that are not easily implemented in most laboratories [30]. To reduce the risk of incorrect determination of *SMN2* copy number, we suggest performing MLPA in both the newborn and the parents. Indeed, MLPA analysis of the family trio, in addition to confirming the screening result, discriminates the *SMN1* genotype and allows the evaluation of whether *SMN2* copy number is consistent between children and parents, thereby supporting the overall results and reducing report times. Nevertheless, in many patients, we were unable to determine the precise position (phases) of *SMN2/NAIP* copies along the 5q13 locus, which could be one of the genomic factors responsible for the variable expressivity of the disease.

A crucial challenge in the genomic NBS era is reporting the carrier status of the participant’s children. In fact, professional guidelines and other opinion papers provided various arguments against performing carrier testing in children [31]. However, there may be benefits to the parents and family members if carrier status identified through NBS was used to aid their own reproductive decision-making, including prenatal diagnosis and therapy [32,33]. Carrier identification in a child may prompt the parents to receive cascade testing to identify their own risk of having an affected child and then potentially take steps to avoid this [30]. In agreement, the British Medical Association has determined that it is “not acceptable to withhold information if it is discovered accidentally” and therefore has advocated for disclosure of this information to the parents [34] (p. 380).

Therefore, while the primary goal of NBS programs is to improve outcomes for the screened babies, our preliminary results indicate that NBS for SMA has the potential to also identify heterozygous carriers of one *SMN1* copy that, based on the disease frequency, are usually unaffected. However, for the very rare heterozygous children who are born or become symptomatic later in life, in-depth genetic investigation could be immediately performed to look for other types of variations in the entire coding region of the *SMN1/2* genes and to confirm or rule out SMA molecular diagnosis more rapidly. The use of long-read-based sequencing represents a promising tool to identify small nucleotide variants, intragenic deletions, and to precisely assess copy number and phase of the SMA 5q-related genes [28].

In conclusion, the validated semi-automated procedure we used for SMA NBS showed excellent performance in detecting homozygous absence of *SMN1* exon 7 and was successful at early identification and treatment of babies with SMA. The current method has limitations when the amplification of the reference gene fails. By overcoming this limitation, the NBS program would implement its diagnostic sensitivity by identifying heterozygous carriers of *SMN1* deletion and consequently increase the detection of families at procreative risk of SMA, with the potential perspective of reducing the incidence and/or severity of this devastating disease in the near future.

## Figures and Tables

**Table 1:** SMA patients identified during the NBS programs and their genotypes.

Patient	Sex	*SMN1*		*SMN2*	*NAIP Exon 5*	c.859G>C
		Allele 1	Allele 2	# Copies	# Copies	
ID.1	F	del	del	2	0	-
ID.2 *	F	del	del	2	0	-
ID.3	F	del	del	2	0	-
ID.4 *	M	del	del	2	0	-
ID.5	F	del	del	2	1	-
ID.6 ^	M	del	del	3	1	-
ID.7 *	M	del	del	3	0	-
ID.8	M	del	del	4	0	+
ID.9	F	del	del	4	1	-
ID.10	F	del	del	4	2	-
ID.11	F	del	c.840C>T	2	1	-
ID.12	F	del	c.840C>T	3	2	-

* Symptomatic newborns; ^ extra-screening. F, female; M, male; del, whole gene deletion.

## Data Availability

The original contributions presented in this study are included in the article. Further inquiries can be directed to the corresponding author.

## Abbreviations

The following abbreviations are used in this manuscript:

SMA	Spinal muscular atrophy
*SMN1*	Survival Motor Neuron 1 gene
*NAIP*	NLR family apoptosis inhibitory protein/BIRC1 gene
NBS	Newborn screening program
DBS	Dried blood spots
IEM	Inborn errors of metabolism
PCR	Polymerase chain reaction
MLPA	Multiplex ligation probe amplification
